# Nutritional psychology and inflammatory bowel disease: a narrative review of gut-brain axis interactions

**DOI:** 10.3389/fnut.2025.1592528

**Published:** 2025-08-01

**Authors:** Omer Horovitz

**Affiliations:** ^1^The Physiology and Behavior Laboratory, Tel-Hai Academic College, Qiryat Shemona, Israel; ^2^Department of Psychology, Tel-Hai Academic College, Qiryat Shemona, Israel

**Keywords:** gut-brain axis, nutritional psychology, psychiatric comorbidities, inflammatory bowel disease, microbiota

## Abstract

This paper explores the intricate relationship between Inflammatory Bowel Disease (IBD) and psychopathology, with a particular focus on anxiety and depression. This narrative review synthesizes recent findings on how dietary factors and nutritional psychology influence the gut-brain axis in patients with inflammatory bowel disease. The bidirectional gut-brain axis, chronic inflammation, and psychological stress are all key contributors to the mental health burden in IBD patients. The paper reviews the physiological mechanisms linking IBD and psychiatric symptoms, particularly how inflammation and gut microbiota composition may influence mood disorders. It addresses the variability in psychiatric comorbidities across IBD subtypes (Crohn’s disease and ulcerative colitis) and highlights the challenges in standardized diagnosis and treatment. Emerging research on microbiome-based therapies, nutritional interventions, and personalized care approaches offers promising solutions for improving gastrointestinal and mental health outcomes. Integrating multidisciplinary care, involving gastroenterologists, psychiatrists, and dietitians, alongside advances in precision medicine, holds potential for developing more effective, individualized treatment strategies. However, challenges remain regarding variability in patient responses, methodological inconsistencies, and the need for standardized clinical protocols. The paper concludes by calling for further research to clarify these relationships and optimize treatment for IBD patients struggling with both physical and psychological health challenges.

## Introduction

Inflammatory Bowel Disease (IBD), which includes Crohn’s disease and ulcerative colitis, refers to chronic gastrointestinal diseases characterized by persistent inflammation of the gastrointestinal tract (1). Recent studies have highlighted an intriguing and growing connection between IBD and psychopathology, particularly psychiatric comorbidities such as anxiety, depression, and other mood disorders (2–4). This relationship has significant implications for patient management and quality of life (5, 6).

Nutritional psychology, an emerging field that examines how diet influences the gut microbiome and mental health, has the potential to play a pivotal role in understanding and addressing these interconnected health challenges [for a rcomperhensive eview, see: Horovitz et al. (7)]. Specifically, the gut-brain axis, the bidirectional communication system linking the gut and brain, is increasingly recognized as a key factor in both IBD and psychiatric disorders. Disruptions in this axis can exacerbate gastrointestinal symptoms and contribute to the development or worsening of psychiatric conditions.

This paper will explore the current understanding of the relationship between IBD and psychopathology, with a particular focus on how dietary factors and the gut microbiome influence both gastrointestinal and psychological symptoms. This review aims to explore the relationship between nutritional psychology and inflammatory bowel disease, focusing on how diet and the microbiome affect both gastrointestinal and psychological outcomes. It will also discuss the research challenges in this field, such as the heterogeneity of IBD and the difficulty in distinguishing causal relationships, and suggest potential avenues for future exploration.

## Methods

This article presents a narrative review of the literature on nutritional psychology and inflammatory bowel disease (IBD). A comprehensive search of PubMed and Google Scholar was conducted using relevant keywords, including *“inflammatory bowel disease,” “nutrition,” “diet,” “psychopathology,” “microbiome,”* and *“gut-brain axis.”* Studies were selected based on their relevance, methodological quality, and recency (published within the past 10 years). The review focused exclusively on human research, excluding case reports and animal-only studies. To ensure transparency and rigor, the review process was guided by the SANRA (Scale for the Assessment of Narrative Review Articles) criteria (8).

## The gut-brain axis and psychopathology in IBD

The relationship between the gut-brain axis and psychopathology is central to understanding how Inflammatory Bowel Disease (IBD) may contribute to psychiatric comorbidities, such as anxiety and depression. The gut-brain axis is a bidirectional communication system that connects the gastrointestinal tract with the central nervous system (CNS), involving pathways such as the Vagus nerve, immune signaling, and gut microbiota, all of which regulate mood, cognition, and behavior (9, 10). The gut, often called the “second brain” due to its extensive network of neurons, can profoundly influence psychological health when disturbed. Disruptions in this axis, caused by chronic gastrointestinal inflammation in IBD, may trigger systemic inflammation, which can, in turn, affect brain function and lead to psychiatric symptoms (11).

## Psychological comorbidities in IBD

Patients with IBD experience higher rates of psychiatric disorders compared to the general population, particularly anxiety and depression. A study found that approximately 25.8% of IBD patients suffer from depression and 21.2% from anxiety, with 30.3% affected by one or both conditions (12). These rates are particularly elevated in patients with Crohn’s disease (CD) and ulcerative colitis (UC). Disease activity, including the severity of inflammation, and factors such as female gender are significantly associated with higher rates of mental health issues in IBD patients. This psychiatric burden is reflected in increased healthcare utilization, with IBD patients visiting primary and emergency care services more frequently and using antidepressants and anxiolytics at higher rates than the general population (13). A recent meta-analysis further confirmed these findings, reporting a significant increase in the prevalence of psychiatric disorders, especially depression and anxiety, in IBD patients (4).

These psychiatric symptoms are often exacerbated during disease flares when patients experience pain, fatigue, and other debilitating IBD-related symptoms (14). Chronic inflammation in the gastrointestinal tract is thought to trigger systemic inflammation, which can affect brain function through neuroimmune mechanisms.

Table 1 summarizes the key relationships between IBD, associated psychopathologies, and the biological mechanisms linking them, including biomarker-related findings.

**Table 1:** Summary of the relationship between inflammatory bowel disease (IBD), psychiatric comorbidities, and associated biological mechanisms including inflammatory and microbiota-related biomarkers.

Domain	Finding	Summary statistic	Source
Depression in IBD	Increased prevalence in IBD patients	25.8% of IBD patients experience depression	Byrne et al. (12)
Anxiety in IBD	Anxiety levels significantly higher than in general population	21.2% of IBD patients affected	Irving et al. (13)
Inflammatory pathways	Pro-inflammatory cytokines contribute to psychiatric symptoms	TNF-α, IL-6, IL-1β elevated in IBD; linked to depression and anxiety via neuroinflammation	Dantzer et al. (47) and Strober (48)
Microbiota-related biomarkers	Gut dysbiosis affects mental health via microbial metabolites and SCFAs	Lower levels of butyrate and serotonin precursors observed in IBD with depressive symptoms	Xiong et al. (49) and Silva et al. (50)

## The role of diet and nutritional psychology

Nutritional psychology is an interdisciplinary field that explores how diet and nutrition impact mental health (7). As such, it is a valuable approach to understanding the connection between Inflammatory Bowel Disease (IBD) and psychopathology. This is especially pertinent in light of the growing body of evidence suggesting that diet plays a critical role in both the pathogenesis of IBD and the modulation of mood and behavior (15, 16).

For instance, a review highlighted that the Mediterranean diet, known for its focus on plant-based foods, healthy fats, and moderate animal product consumption, was associated with a reduced risk of chronic diseases, including IBD (17). Another review reported similar favorable effects of the diet, suggesting that it supports gut barrier function and reduces inflammation. However, adherence to this diet among IBD patients is often low (18). Nevertheless, recent studies suggest that adherence to the Mediterranean diet can improve significantly when patients receive proper support, such as nutritional counseling and tailored interventions. Supportive programs, particularly those incorporating individualized meal planning and education on the benefits of the diet, have increased adherence and provided better outcomes for IBD and mental health symptoms (19, 20).

In contrast, a high-fat and high-sucrose diet significantly contributes to the onset of IBD (21). Additionally, the rising prevalence of IBD has been linked to high consumption of ultra-processed foods (UPFs), which are rich in fats, sugars, salt, and additives that disrupt the intestinal barrier (22). These findings underscore the importance of dietary choices in managing IBD, suggesting that promoting adherence to diets like the Mediterranean diet while minimizing consumption of the Western diet could help mitigate the risk and progression of the disease.

A parallel body of research has examined the impact of the Mediterranean diet on mental health, particularly anxiety, stress, and depression. One study found that higher adherence to the Mediterranean diet was inversely associated with symptoms of anxiety and stress, but not depression, in older adults in Australia (23). Another randomized controlled trial showed that after 12 weeks, participants following the Mediterranean diet experienced significant reductions in anxiety, stress, and depression levels, along with improvements in weight and BMI, though the clinical significance was limited (24). A recent review further indicated that adherence to the Mediterranean diet is associated with a reduced risk of depressive symptoms, with substantial evidence supporting its link to a lower incidence of depression in prospective studies (25). However, it is essential to note that the relationship between diet quality and clinical depression or anxiety remains inconsistent. While some dietary interventions show promise, further high-quality studies are needed to establish more explicit conclusions and clinical recommendations. Conversely, a Western diet has been associated with higher psychological distress (26), and another study found a higher risk of depression and anxiety associated with a Western diet (27). Similarly, high intake of processed foods and sugar and low vegetable consumption have been linked to increased depressive and anxiety symptoms (28). In addition to the Mediterranean diet, other dietary patterns, such as the DASH diet (Dietary Approaches to Stop Hypertension), which emphasises fruits, vegetables, whole grains, and lean proteins, have also shown promise in reducing symptoms of depression and improving mental well-being (29). Together, while the Mediterranean diet shows promising potential in reducing anxiety, stress, and depressive symptoms, further rigorous studies are necessary to clarify its clinical significance and establish definitive guidelines for its role in mental health management.

Although research on the Mediterranean diet’s effects on IBD and mental health has typically been conducted separately, with studies focusing on gut health and psychopathology in isolation, the emerging field of nutritional psychology offers a unique opportunity to bridge this gap. Nutritional psychology can integrate findings from both areas by highlighting the connection between gut health, dietary patterns, and psychological well-being. This interdisciplinary approach offers a more holistic understanding of how diet influences both physical and mental health, providing more comprehensive strategies for managing conditions like IBD and mental health disorders simultaneously.

Several nutritional interventions have been explored in the context of IBD and mental health, shedding light on the intricate connections between diet, gut health, and psychological well-being. Emerging evidence suggests that nutritional interventions are pivotal in addressing these interconnected challenges.

Omega-3 fatty acids, particularly those derived from fish oil, are among the most studied nutritional interventions (30). Known for their potent anti-inflammatory effects, omega-3 s can directly modulate cytokine activity, reducing the systemic inflammation that drives IBD symptoms (31, 32). Beyond their impact on inflammation, omega-3 s are also recognized for their psychotropic effects (33). By enhancing synaptic plasticity and modulating neurotransmitter systems such as serotonin and dopamine, omega-3 s may reduce depressive symptoms and improve emotional regulation (34, 35). These dual-action benefits make omega-3 s a compelling dietary addition for individuals with IBD, addressing both physical inflammation and associated mental health challenges.

Cutting-edge research further highlights the role of personalized nutrition in managing IBD and its psychiatric comorbidities. Advances in microbiome sequencing and metabolomics now enable tailored dietary interventions that address individual variations in gut microbial composition and metabolic responses. For example, targeting specific microbial imbalances with customized probiotics or prebiotics may offer more precise therapeutic outcomes (36, 37). Moreover, the integration of psychobiotics, live microorganisms with documented effects on mental health, represents an innovative approach to addressing both IBD and its psychological dimensions (38).

The idea that nutritional strategies can target both the gut and the brain is transforming the management of chronic conditions like IBD. These interventions not only address the physical symptoms of IBD but also provide a complementary, non-pharmacological avenue for improving mental health. These findings suggest that dietary strategies, particularly those that target inflammation and support microbiota diversity, may improve both gastrointestinal and psychological outcomes in IBD. Table 2 provides a comparative overview of major nutritional interventions, their effects on IBD symptoms, and their potential impact on mental health.

**Table 2:** Summary of selected nutritional interventions and their reported impacts on inflammatory bowel disease (IBD) progression and psychiatric symptoms (depression and anxiety).

Intervention	Type of evidence	Effect on IBD	Effect on mental health	Key studies
Mediterranean diet	RCTs, Meta-analysis	↓ Inflammation; ↑ Barrier Function	↓ Depression and Anxiety (inconsistent)	Eliby et al. (25) and Parletta et al. (59)
Omega-3	Review, RCTs	↓ CRP, TNF-α	↑ Serotonin modulation	Grosso et al. (34)
Probiotics (e.g., Bifidobacteria)	RCTs	Gut flora restoration	↓ Depression scores	Merkouris et al. (44)

As research in this field advances, incorporating personalized, microbiome-informed dietary strategies could redefine how we approach the treatment of IBD and its psychiatric comorbidities, highlighting the profound interplay between nutrition, the gut, and the mind. Nutritional interventions underscore the intricate connection between gut health and mental well-being in managing IBD and its psychiatric comorbidities. These strategies demonstrate the transformative potential of nutrition in managing the gut-brain axis in IBD despite challenges in understanding the complex, bidirectional dynamics involved. Table 2 presents nutritional interventions in managing IBD and psychopathology.

## Microbiome and dietary modulation

Gut microbiota is crucial in modulating brain function and behavior. Alterations in microbiota composition, a phenomenon known as dysbiosis, have been observed in individuals with IBD and are also linked to psychiatric symptoms.

The growing understanding of the gut-brain axis has sparked increased interest in nutritional psychology, which examines how dietary factors influence mental health. Diet plays a central role in shaping the gut microbiome, with fibre-rich and fermented foods promoting beneficial bacteria. In contrast, highly processed and high-sugar diets are associated with dysbiosis and inflammation (39). Specific dietary interventions, such as the Mediterranean diet or probiotics, have shown promise in modulating gut microbiota and improving gastrointestinal and mental health outcomes (40). For individuals with IBD, nutritional strategies targeting the microbiome may offer a complementary approach to managing inflammation and associated psychiatric symptoms, paving the way for integrated treatment paradigms.

Another area of significant interest is the role of probiotics, prebiotics, and dietary fibre in modulating gut microbiota. Probiotics, which introduce beneficial bacteria, and prebiotics, which serve as fuel for these microbes, have demonstrated promise in restoring microbial balance and alleviating gastrointestinal and psychological symptoms (36, 41, 42). Emerging research suggests that specific strains, such as *Lactobacillus* and *Bifidobacterium*, may be particularly effective in modulating mood and reducing symptoms of depression and anxiety by influencing the production of short-chain fatty acids and anti-inflammatory metabolites (43, 44). Furthermore, recent studies have indicated that increasing dietary fibre, particularly from sources like whole grains, legumes, and fruits, can enhance gut health and potentially alleviate symptoms of anxiety and depression through microbiome modulation (45).

## Mechanisms linking nutrition to psychopathology

Pro-inflammatory cytokines, such as tumor necrosis factor-alpha (TNF-*α*), interleukin-6 (IL-6), and interleukin-1β (IL-1β), are implicated in both the pathogenesis of IBD and the onset of psychiatric disorders, suggesting that systemic inflammation may underlie the comorbidity of these conditions. These cytokines can activate neuroimmune cells in the brain, disrupt the blood–brain barrier, and trigger neuroinflammation, all of which can contribute to psychiatric symptoms such as depression and anxiety (46–48).

Dysbiosis can disrupt the production of key metabolites, such as short-chain fatty acids (SCFAs) and neurotransmitters like serotonin, essential for both gastrointestinal and psychological health (49, 50). In IBD patients, disruptions in the microbiome may alter the production of these molecules, contributing to the onset or worsening of psychiatric symptoms (51, 52). These findings emphasize the intricate connection between the gut microbiota, immune system, and CNS, underscoring the potential of microbiome-targeted therapies to alleviate gastrointestinal and psychiatric symptoms in IBD patients.

SCFAs such as butyrate, propionate, and acetate are produced through the fermentation of dietary fibers by gut microbiota (53, 54). These metabolites have anti-inflammatory and neuroprotective effects (55). SCFAs help maintain blood–brain barrier integrity, reduce neuroinflammation, and influence neurotransmitter synthesis, factors critically linked to mood regulation and cognitive health (50, 56). Diets rich in prebiotics and fermentable fibers may increase SCFA levels (57), thereby mitigating psychiatric symptoms often seen in IBD patients.

## Dietary patterns and brain function

As noted previously, the Mediterranean diet has been consistently associated with reduced risk of depression, anxiety, and cognitive decline (58). Its nutrient composition supports gut microbial diversity and reduces systemic inflammation, both of which are relevant for mental health. Importantly, this diet also promotes brain-derived neurotrophic factor (BDNF), which enhances neuroplasticity and may contribute to resilience against stress-related disorders (59).

In contrast, the Western diet as been linked to increased rates of psychological distress, including depression and anxiety (2, 27). This pattern promotes systemic inflammation, oxidative stress, and dysbiosis, all of which can compromise brain function and emotional regulation (60).

Dietary patterns influence brain function through multiple biological pathways. Pro-inflammatory diets can increase circulating cytokines, such as IL-6 and TNF-*α*, which affect mood and cognition by promoting systemic and neuroinflammation (61). These inflammatory responses are well-documented contributors to the development of depression and anxiety (62, 63).

Additionally, nutrients like tryptophan and folate play a crucial role in the biosynthesis of key neurotransmitters, including serotonin and dopamine (64). Serotonin, which is predominantly produced in the gut, is essential for regulating mood (65). Inadequate intake of these precursors can impair neurotransmitter production and contribute to the onset or worsening of depressive symptoms (66).

Together, these findings underscore that dietary patterns such as the Mediterranean or Western diets can significantly impact psychological outcomes in IBD patients by modulating inflammatory responses, gut microbiota composition, and neurochemical signaling. Diets abundant in prebiotics and fermentable fibers have been shown to elevate SCFA production (67), offering a promising strategy to alleviate psychiatric symptoms commonly observed in patients with IBD.

## Current gaps and future directions

Despite the growing recognition of the link between Inflammatory Bowel Disease (IBD) and psychopathology, several challenges remain in understanding the precise mechanisms underlying this relationship. A comprehensive examination of the psychological implications of IBD necessitates consideration of the social, emotional, and psychological burden on patients. The differences between Crohn’s disease and ulcerative colitis extend beyond the gastrointestinal tract, influencing the patient’s overall experience and psychological response. Crohn’s disease is more likely to cause complications such as strictures and fistulas, which may result in heightened stress and exacerbation of psychiatric symptoms due to the chronic and unpredictable nature of the disease (68). In contrast, ulcerative colitis is generally confined to the colon, often leading to a more predictable disease course and psychological impact (69, 70). This disparity emphasizes the necessity of personalized treatment protocols that consider both the gastrointestinal and psychological manifestations of IBD. This variability impacts how patients experience the disease and its associated psychological effects, making it difficult to identify universal patterns.

Scientific evidence supports the notion that chronic inflammation in IBD contributes to changes in the central nervous system (CNS) (71), potentially driving psychiatric symptoms. This suggests a biological basis for the overlap between IBD and psychopathology, indicating that inflammation may directly influence the development of psychiatric symptoms. However, further research is needed to clarify the specific neuroinflammatory pathways involved and how these can be targeted for therapeutic benefit.

While chronic stress from living with IBD may exacerbate psychiatric symptoms through the dysregulation of the HPA axis, emerging research suggests that chronic inflammation may also contribute directly to psychiatric outcomes. This dual pathway underscores the need for a nuanced understanding where both stress and inflammation may interact to amplify psychiatric comorbidities in IBD patients (72, 73). Further, the role of psychological resilience and coping strategies in modulating these pathways remains an area of great potential for intervention.

The difficulty in establishing causality is compounded by the variability in psychiatric comorbidities among individuals with IBD. Anxiety and depression are the most frequently reported, with prevalence rates significantly higher than in the general population (2, 12). However, other psychiatric conditions, such as bipolar disorder and post-traumatic stress disorder (PTSD), have also been observed, albeit less commonly (4, 74). This spectrum of mental health challenges underscores the need for personalized approaches to understanding and treating the psychiatric comorbidities of IBD. It is essential to consider how these comorbidities may interact with each other and impact treatment strategies, as patients may experience overlapping mental health issues. A summary of the main challenges in understanding the IBD-psychopathology relationship, including their implications for research and treatment, is presented in Table 3.

**Table 3:** Key Challenges in Understanding the Relationship Between Inflammatory Bowel Disease (IBD) and Psychopathology, with Implications for Research and Clinical Practice.

Challenge	Description	Implication for research and clinical practice	Citation
IBD subtype variability	Differences between Crohn’s and UC in clinical course and psychological burden	Research must stratify by subtype; treatments should be disease-specific	Le Berre et al. (68)
Dual pathways (Stress and Inflammation)	Chronic stress and inflammation both contribute to psychiatric symptoms	Interventions should target both psychological and inflammatory processes	Peppas et al. (73)
Lack of standardized diagnostic tools	No consistent psychiatric screening or diagnostic criteria in IBD populations	Need to develop validated, disease-contextualized mental health screening tools	Singh et al. (83)
Heterogeneity of psychiatric comorbidities	Patients may present with a range of disorders beyond anxiety/depression (e.g., PTSD, bipolar)	Personalized assessment and treatment plans are needed	Taft et al. (74)
Overlap of somatic and psychological symptoms	Symptoms like fatigue and pain may obscure psychiatric diagnosis	Improve clinical tools to distinguish physical vs. mental symptomatology	Peppas et al. (73)
Need for longitudinal studies	Current research is mostly cross-sectional, limiting causal inference	Long-term studies can identify temporal patterns and predictive factors	Lakhan and Kirchgessner (71)
Multidisciplinary approach needed	Fragmented care misses links between gut and mental health	Collaborative models can improve holistic outcomes for IBD patients	Riggott et al. (98)

Furthermore, the lack of standardized diagnostic criteria for psychiatric comorbidities in IBD patients presents another significant obstacle. While anxiety and depression are commonly assessed using self-report measures, there is no consensus on the most effective tools for evaluating psychiatric symptoms in the context of chronic illness. Developing and validating tools designed explicitly for assessing mental health in IBD patients will be critical in improving the accuracy of diagnoses and tailoring treatments. This inconsistency in assessment methods makes it difficult to compare findings across studies. Moreover, self-report measures may not fully capture the complexity of psychiatric symptoms in IBD patients, as they often fail to account for the overlap between somatic and psychological symptoms, such as fatigue and pain, which are common in both IBD and depression.

Longitudinal studies that track the onset and progression of both gastrointestinal and psychiatric symptoms over time can provide valuable insights into the relationship between IBD and psychopathology. Such studies would allow researchers to identify temporal patterns and potential causative factors, shedding light on whether psychiatric symptoms precede, coincide with, or follow IBD flare-ups. Recent advances in big data analytics and real-time monitoring offer exciting possibilities to capture this temporal variability more comprehensively. Advances in neuroimaging techniques and biomarker identification can also contribute to understanding the mechanisms linking IBD and mental health. For instance, functional magnetic resonance imaging (fMRI) studies have revealed altered brain connectivity in IBD patients, particularly in regions associated with mood regulation and stress response, providing further evidence of the gut-brain connection (75–77).

Addressing these challenges will require a multidisciplinary approach, combining gastroenterology, psychiatry, and immunology expertise. Efforts to foster collaboration between researchers across disciplines are essential in generating comprehensive insights into the IBD-psychopathology link. Integrating psychological screening into routine IBD care can help identify at-risk patients early and facilitate timely intervention. Moreover, future research should prioritize the development of standardized diagnostic criteria and evidence-based treatment strategies for psychiatric comorbidities in IBD. By unraveling the complex interplay between IBD and psychopathology, researchers and clinicians can improve outcomes for patients grappling with both physical and mental health challenges.

## Scientific progress and future research directions

The future of research on IBD and psychopathology is promising, with several emerging areas that may shed light on the complex relationship between these two domains. To fully realize the therapeutic potential of these emerging areas, research should focus on establishing rigorous, randomized controlled trials with larger, more diverse patient populations. Advances in microbiome research offer exciting possibilities for understanding how gut health influences mental health. Studies exploring the use of microbiota-based therapies, such as fecal microbiota transplantation (FMT) or specific probiotic strains, may provide novel treatments for both IBD and its psychiatric comorbidities (78, 79). For example, alterations in gut microbial composition have been associated with increased levels of anxiety and depression in IBD patients, highlighting the therapeutic potential of targeting the microbiome (80). However, the use of microbiome-based interventions in clinical practice is still in its early stages, and more research is needed to determine the safety and efficacy of these therapies. Some key issues lie in the differences between Crohn’s disease and ulcerative colitis (UC). While fecal microbiota transplantation has shown effectiveness in several randomized controlled trials for UC, it has shown no benefit in Crohn’s disease (81). Furthermore, future research should focus on uncovering the mechanisms that drive differential responses to microbiome-based interventions in these two subtypes (82).

Further studies in nutritional psychology are essential to identify specific dietary patterns or nutrients that may help mitigate the psychological burden of IBD. Diet plays a crucial role in shaping the gut microbiome and influencing inflammation, a key gastrointestinal and mental health factor. Incorporating precision nutrition into IBD care may offer a personalized approach to managing both the physical and psychological aspects of the disease. Randomized controlled trials (RCTs) evaluating the impact of dietary interventions on gastrointestinal and psychiatric outcomes will be essential in establishing evidence-based guidelines for dietary management in IBD patients. Additionally, using nutrition screening tools in research and practice could significantly enhance understanding the relationship between diet and mental health in IBD. Assessing the prevalence of malnutrition risk in patients with IBD using designated screening tools emphasized the importance of nutritional assessment for improving clinical outcomes and addressing malnutrition and sarcopenia in IBD patients (83, 84).

Anti-TNF therapy, commonly used to manage IBD, has also shown benefits for mental health in many patients. Studies have reported significant reductions in anxiety and depression symptoms following anti-TNF therapy, likely due to the reduction in inflammation and improvement in disease control (85–87). This highlights the potential role of anti-TNF therapy not only in managing the physical symptoms of IBD but also in improving the psychological well-being of patients. However, the long-term mental health outcomes of such treatments remain understudied and warrant further investigation. However, the long-term mental health outcomes of such treatments remain understudied and warrant further investigation. The effects may vary across individuals, and further investigation is needed to understand the long-term mental health benefits of these therapies.

Individual variability in response to dietary and microbiome interventions is a critical factor that underlines the importance of precision nutrition in managing conditions like IBD and its associated mental health comorbidities. Recent advances in microbiome research have shown that genetic, environmental, and lifestyle factors contribute to variations in how individuals respond to dietary interventions. For example, specific individuals may experience significant improvements in gut health and psychological well-being with the introduction of probiotics (88). In contrast, others may show minimal response due to differences in their baseline microbiome composition or genetic predispositions (89, 90). Similarly, dietary interventions such as the Mediterranean diet may be more effective for some individuals, depending on their metabolic profiles, gut microbiota, and underlying health conditions (91).

In contrast, others may not see the same benefits (92). This individual variability suggests that a one-size-fits-all approach to nutrition may not be suitable for everyone and highlights the need for personalized dietary strategies that consider an individual’s unique microbiome and genetic makeup. Personalized dietary interventions may, in the future, become an integral part of IBD care, improving both disease outcomes and mental health. Precision nutrition, therefore, involves tailoring dietary recommendations to optimize health outcomes based on personal biomarkers, microbiome sequencing, and metabolic profiling (93, 94). By incorporating these personalized approaches, healthcare providers can better address the complex interplay between diet, microbiome, and mental health in IBD patients, ultimately leading to more effective and targeted treatments.

To further support the integration of nutrition and mental health, combining nutrition strategies with psychological treatments could offer a comprehensive approach for managing both physical and psychological symptoms in IBD patients. Cognitive-behavioral therapy (CBT) and mindfulness-based stress reduction (MBSR) have demonstrated effectiveness in reducing psychological distress in IBD patients (95), and these interventions can be complemented by specific dietary strategies designed to reduce inflammation and support gut health. For instance, mindfulness-based eating practices could be integrated into dietary interventions to promote healthier food choices and reduce stress. At the same time, cognitive-behavioral strategies could help patients address food-related anxieties that may exacerbate their symptoms (96). Future studies should explore the synergistic effects of these combined approaches, evaluating their impact on gastrointestinal and mental health outcomes. The integration of psychological therapies with nutritional interventions could optimize the overall well-being of patients with IBD and reduce the need for separate treatments for the gut and mental health aspects of the disease.

Better clinical integration of mental health care in the treatment of IBD is also needed. Collaborative care models, in which gastroenterologists, psychologists, and dietitians work together to address IBD patients’ physical and mental health needs, can improve overall patient outcomes. Patients receiving integrated care reported significant improvements in mental health and disease activity (97–99). Developing standardized protocols for assessing and managing psychiatric symptoms in IBD patients would be a key step in achieving this integration. Routine mental health screenings, combined with evidence-based interventions such as cognitive-behavioral therapy (CBT) and mindfulness-based stress reduction (MBSR), can help reduce the psychological burden of IBD.

Additionally, personalized medicine holds great promise for advancing the field. Advances in genomics and biomarkers may enable the identification of individuals at higher risk for developing psychiatric comorbidities in the context of IBD (100). For instance, genetic studies have linked specific polymorphisms in inflammatory pathways, such as variations in the IL-1β and TNF-*α* genes, to both IBD severity and susceptibility to depression (101, 102). By tailoring treatments based on an individual’s genetic and microbiome profile, clinicians may be able to offer more effective and targeted interventions.

The integration of telenutrition and telemedicine into IBD management represents a growing area of interest, offering potential solutions for addressing barriers to access and improving multidisciplinary care. Telenutrition, which involves remote dietary counseling and monitoring, can complement existing models by facilitating collaboration between gastroenterologists, psychiatrists, and dietitians. Research indicates that telemedicine interventions may not necessarily improve depressive symptoms or overall quality of life compared to standard care (103). However, meta-analyses suggest that telemedicine has a role in optimizing disease management and symptom monitoring (104). Additionally, emerging literature highlights both the benefits and limitations of telenutrition in IBD care, particularly in personalized dietary guidance and long-term adherence (105). Future research should explore how telenutrition can be integrated effectively into multidisciplinary care models to enhance both physical and mental health outcomes in IBD patients with psychiatric comorbidities.

Integrating cutting-edge technologies like machine learning and artificial intelligence (AI) can further enhance our understanding of the IBD-psychopathology relationship. AI-driven analyses of large datasets, including electronic health records and patient-reported outcomes, can uncover previously unrecognized patterns and predictors of psychiatric comorbidities. This data-driven approach can also aid in identifying optimal treatment strategies and monitoring disease progression in real time. Table 4 summarizes the emerging research areas that hold promise for advancing the understanding and treatment of IBD and its psychiatric comorbidities, along with their clinical potential, key challenges, and current evidence base.

**Table 4:** Emerging research areas in IBD-psychopathology: description, clinical potential, challenges, and evidence status.

Research area	Description	Clinical potential	Key challenges	Evidence level	Key references
Microbiome-based therapies	FMT and probiotics to alter gut-brain interactions	Dual impact on IBD and mental health	Subtype variability (Crohn’s vs. UC), safety, early clinical stage	Early-stage trials	Mayer et al. (78)
Nutritional psychology	Role of diet (e.g., Mediterranean) in shaping mental and gut health	Accessible non-pharmacologic strategy	Lack of standardization, need for RCTs	Observational + RCTs	Horovitz. (7)
Precision nutrition	Tailored diets based on microbiome/genetics	Targeted treatments, improved adherence	High cost, limited clinical application so far	Emerging research	Singh et al. (52)
Psychological + nutritional integration	Combining CBT/MBSR with dietary interventions	Holistic, patient-centered treatment	Synergistic effects not well documented	Early-phase studies	Brewer et al. (96)
Collaborative care models	Unified gastro-psych-dietitian model for IBD care	Multidimensional improvement in quality of life	Lack of implementation protocols, training gaps	Meta-analysis, RCTs	Riggott et al. (98)
Telemedicine/telenutrition	Remote care delivery and monitoring	Expanded access, especially in underserved areas	Mixed evidence on mental health, adherence difficulties	Mixed-methods data	Pang et al. (104)
Machine learning/AI	Big data and predictive modeling for psychiatric risk	Data-driven precision in diagnosis and treatment planning	Algorithm bias, data privacy, limited IBD-specific models	Exploratory	Felger et al. (61)
Biomarker identification	Inflammatory and genetic indicators for psychiatric comorbidity risk	Enables early intervention and precision treatment	Needs validation, unclear mechanistic pathways	Pilot and correlation studies	Das et al. (102)

In conclusion, the future of research on IBD and psychopathology is marked by exciting opportunities and challenges. Advances in microbiome science, nutritional psychology, collaborative care models, and personalized medicine offer promising avenues for improving patients’ lives affected by both conditions. To fully realize this potential, sustaining investment in multidisciplinary research and developing standardized clinical protocols will be essential.

## Limitations

While this review provides a comprehensive overview of the gut-brain axis, nutritional psychology, and interventions targeting IBD-related psychopathology, several limitations must be acknowledged. First, IBD is a highly heterogeneous condition with varying phenotypes, disease severities, and treatment responses that complicate generalizations. Second, psychiatric symptom assessment is not standardized across studies, leading to variability in reported outcomes. Dietary intervention studies are notoriously difficult to conduct due to factors like variability in diet, small sample sizes, and differing methodologies. Additionally, the small number of well-designed trials suggests benefits in Crohn’s but not UC, further complicating the ability to generalize findings across IBD subtypes. Addressing these challenges in future research through more uniform methodologies and personalized treatment approaches will be essential for drawing more definitive conclusions.

As a narrative review, this study is limited by potential selection bias in study inclusion and lacks formal risk-of-bias assessment. The absence of quantitative synthesis and heterogeneity among included studies may limit generalizability.

## Conclusion

The relationship between diet, the microbiome, and mental health in patients with inflammatory bowel disease (IBD) is an increasingly important focus of research with the potential to transform clinical management. Current evidence supports the therapeutic value of targeted nutritional interventions, particularly those that enhance microbiome health and reduce systemic inflammation. However, the extent to which dietary changes alone can improve psychiatric outcomes remains uncertain, underscoring the need for more robust longitudinal and interventional studies.

As the field of personalized medicine evolves, integrating genomic data, microbiome profiles, and lifestyle factors may provide a more individualized framework for managing the physical and psychological complexities of IBD. In parallel, the incorporation of telenutrition and multidisciplinary care models holds promise for delivering more accessible and coordinated interventions. Still, challenges such as disease heterogeneity, variability in psychiatric symptoms, and the absence of standardized diagnostic tools must be addressed.

Ultimately, a nuanced and integrative approach, drawing from advances in nutritional psychology, microbiome science, and personalized nutrition, may be key to optimizing treatment outcomes for IBD patients. Continued interdisciplinary collaboration and high-quality research will be critical for developing tailored interventions that address both gastrointestinal and mental health dimensions of this complex condition.
